# Factors influencing pharmacological treatment in COPD: a comparison of 2005 and 2014

**DOI:** 10.1080/20018525.2017.1409060

**Published:** 2017-12-04

**Authors:** Josefin Sundh, Joakim Åberg, Mikael Hasselgren, Scott Montgomery, Björn Ställberg, Karin Lisspers, Christer Janson

**Affiliations:** ^a^ Department of Respiratory Medicine, School of Medical Sciences, Örebro University, Örebro, Sweden; ^b^ School of Medical Sciences, Örebro University, Örebro, Sweden; ^c^ Clinical Epidemiology and Biostatistics, School of Medical Sciences, Örebro University, Örebro, Sweden; ^d^ Clinical Epidemiology Unit, Department of Medicine, Karolinska Institutet, Stockholm, Sweden; ^e^ Department of Epidemiology and Public Health, University College, London, UK; ^f^ Department of Public Health and Caring Sciences, Family Medicine and Preventive Medicine, Uppsala University, Uppsala, Sweden; ^g^ Department of Medical Sciences: Respiratory; Allergy and Sleep Research, Uppsala University, Uppsala, Sweden

**Keywords:** LAMA, LABA, ICS, bronchodilator therapy, triple inhaled therapy, symptoms, frequent exacerbations GOLD 2017

## Abstract

**Introduction**: The aim was to investigate how the pattern of pharmacological treatment in Swedish patients with chronic obstructive pulmonary disease (COPD) has changed over a decade, and to identify factors associated with treatment.

**Methods**: Data on patient characteristics and pharmacological treatment were collected using questionnaires from two separate cohorts of randomly selected primary and secondary care patients with a doctor’s diagnosis of COPD in central Sweden, in 2005 (*n* = 1111) and 2014 (*n* = 1329). Cross-tabulations and chi-square tests were used to compare maintenance treatment in 2005 and 2014, and to investigate the distribution of treatment by the 2017 Global Initiative for Obstructive Lung Disease (GOLD) ABCD groups. Multinomial logistic regression was used to analyze associations with the major types of recommended treatments: bronchodilator therapy, combined long-acting beta-2-antagonists (LABA) + inhaled corticosteroids (ICS), and triple inhaled therapy.

**Results**: The proportion of patients with no maintenance treatment, with only LABA + ICS, and with sole ICS statistically significantly decreased (36 vs. 31%, 16 vs. 12% and 5 vs. 2%, respectively), and the proportion with triple inhaled therapy statistically significantly increased (29 vs. 40%). In 2014, triple inhaled therapy was the most common treatment in all GOLD groups except group A. In 2014, previous frequent exacerbations [OR (95% CI) 2.34 (1.62 to 3.36)], worse COPD Assessment Test score [1.07 (1.05 to 1.09)], female sex [2.13 (1.56 to 2.91)], and access to a specific responsible doctor [1.95 (1.41 to 2.69)] were associated with triple inhaled therapy. Current smoking [0.40 (0.28 to 0.57)] and overweight [0.62 (0.41 to 0.93)] were inversely associated with triple inhaled therapy.

**Conclusions**: Over the last decade, triple inhaled therapy has increased, and no maintenance treatment, ICS, or LABA + ICS has decreased. Triple inhaled therapy is the most common treatment and is associated with previous exacerbations, higher symptom level, female sex, and having a specific responsible doctor.

## Introduction

Traditionally, severity assessment in chronic obstructive pulmonary disease (COPD) has been based on lung function, but since 2011, the Global Initiative for Obstructive Lung Disease (GOLD) recommendations for assessment of disease severity have also included exacerbation frequency and symptom evaluation. In the updated 2017 version, a distinction has been made between spirometric staging (stage I–IV) and risk assessment based solely on frequent exacerbations and a high level of symptoms (group A–D) [1]. Frequent exacerbations are still defined as two or more exacerbations or one hospitalized exacerbation during the previous year. Symptoms can be evaluated using the COPD Assessment Test (CAT) [2], Clinical COPD Questionnaire (CCQ) [3], or Modified Medical Research Council (mMRC) scale [4].

**Figure 1.**
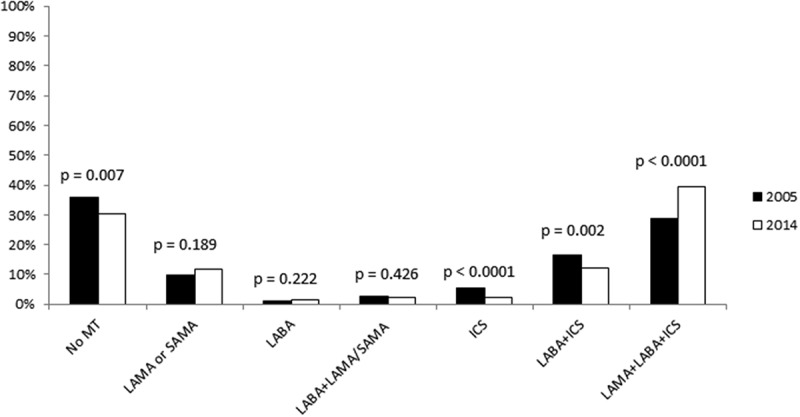
Treatment patterns 2005 and 2014. Treatment with different medicines or combinations, in 2005 compared with 2014. MT = maintenance treatment; LAMA = long-acting muscarinic antagonists; SAMA = short-acting muscarinic antagonists; LABA = long-acting beta-2-agonists; ICS = inhaled corticosteroids.

**Figure 2.**
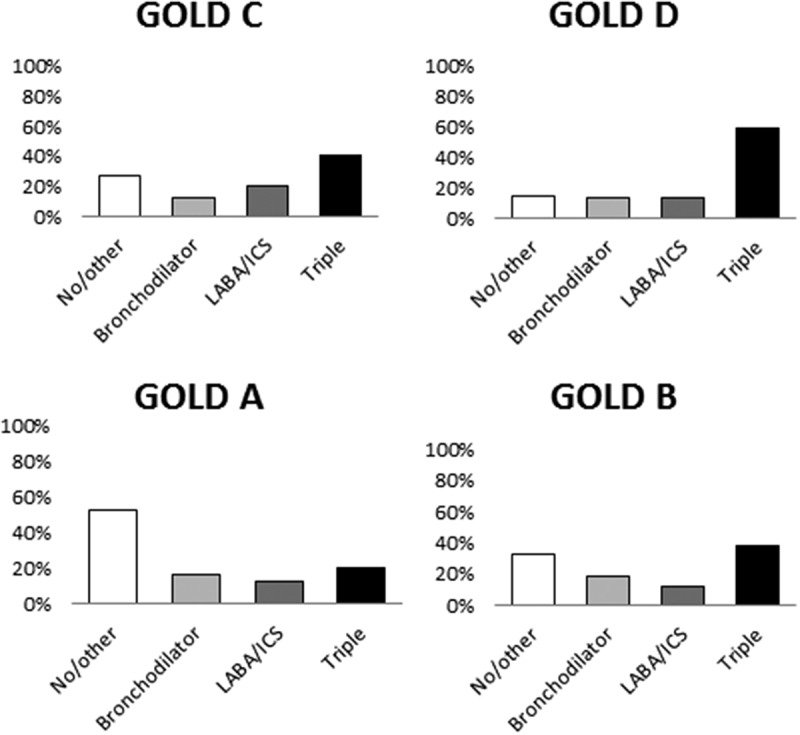
Major treatments in 2014 according to GOLD 2017 groups. Proportion of the total study population distributed over main treatment groups and GOLD ABCD groups. Bronchodilator therapy includes maintenance treatment with long- or short-acting muscarinic antagonists and/or long-acting beta-2-agonists. LABA = long-acting beta-2-agonists; ICS = inhaled corticosteroids. No/other includes patients with only rescue medication and with only ICS.

Pharmacological treatment in COPD today principally includes long-acting muscarinic antagonists (LAMA), long-acting beta-2-agonists (LABA), and inhaled corticosteroids (ICS). Previously, anticholinergic therapy was also prescribed as maintenance treatment with short-acting muscarinic antagonists (SAMA) three or four times daily. There is extensive scientific knowledge of the treatment effects of LAMA or LABA therapy as well as the combinations LAMA + LABA, LABA + ICS, and triple inhaled therapy with LAMA, LABA, and ICS, on exacerbation frequency and health-related quality of life [5–8]. However, previous studies have shown that the prescription pattern differs and does not always follow guidelines [9–16].

The aim of this study was to investigate how the pattern of pharmacological treatment in Swedish primary and secondary care patients with COPD has changed over a decade, and how different factors are associated with prescription of recommended treatments.

## Methods

### Data collection

In 2005, the PRAXIS study COPD cohort was created, with primary and secondary care patients from seven county councils in central Sweden [17–20]. Each county council was represented by the department of respiratory medicine in their central hospital, the department of internal medicine from one randomly selected district hospital and eight randomly selected primary health care centers (PHCCs), in a total of 14 hospitals and 56 PHCCs. In Sweden, patients managed at the PHCCs have a responsible general practitioner, and patients managed at hospitals have responsible specialists in internal or respiratory medicine. A list of all adult patients up to 75 years with a doctor’s diagnosis of COPD (ICD-10 code J44) in the medical records during the period of 2000–2003 was compiled for every participating center. A centralized random selection recruited 1089 patients, including 775 in primary care and 314 in hospital clinics. In 2014, a new random selection of COPD patients was performed at the same 14 hospitals and at 54 of the original 56 PHCCs, recruiting 1329 patients (893 from primary care and 436 from hospitals). In both 2005 and 2014, data were collected using patient questionnaires. The 2014 questionnaire was somewhat extended compared with the 2005 questionnaire. The data collection in 2005 also included record reviews and found that spirometry data were not often performed during the study period [21]. As the aim of the present study was to perform a real-life investigation of patients with a doctor’s diagnosis of COPD to reflect the actual pharmacological treatment of COPD according to the GOLD 2017 ABCD groups, we chose to use only questionnaire data here.

All data in the questionnaires were self-reported, and the questionnaires were posted to the patients and returned in a prestamped envelope. The items of maintenance treatment were expressed to ask if the patient used a specific type of medication during the previous 6 months, with the response alternatives ‘Yes, regularly’, ‘Yes, in periods’, ‘No’, or ‘I do not know’. In our analyses, use of maintenance treatment was defined as self-reported regular use. The items asked for the specific use of LAMA, LABA, ICS, and LABA/ICS respectively by spelling out the names of available sales names. No pictures were used in the questionnaires. At the time of the two data collections, there were still a limited selection of different inhaled COPD medications available in Sweden, no firm combinations of LAMA and LABA had yet arrived in the market, and thus listing of all different sales names within each item was possible.

### Patient characteristics and measures

Patient questionnaires provided data on current pharmacological treatment, sex, age, level of education, smoking habits, body mass index (BMI), number of exacerbations during the previous 6 months, the CCQ score, and, as a marker for continuity of care, awareness of a specific doctor responsible for the COPD treatment. From the 2014 questionnaire, additional data on comorbid diagnoses of heart disease, depression/anxiety, diabetes, and the CAT score were obtained. The dichotomous educational variable identified the most highly educated group as those who had continued in full-time education for at least 2 years beyond the Swedish compulsory school period of 9 years. Smoking habits were categorized as current daily smoking or not. BMI was calculated from self-estimated length and weight, and categorized as underweight (BMI <20), normal weight (BMI 20–24), overweight (BMI 25–29), and obesity (BMI ≥30). Exacerbations were defined as emergency visits (primary care visits, outpatient visits, or hospitalization) in primary or secondary care due to deterioration in lung disease, or need for an oral steroid course, in the recent 6 months. At the time of the first questionnaire, the present GOLD ABCD groups based on annual exacerbations frequency were still not created and implemented, and the choice of asking for exacerbations in the previous 6 and not 12 months was made to decrease the risk for recall bias. Frequent exacerbations were defined here as having one or more exacerbations during the previous 6 months. The CCQ score was dichotomized as high (CCQ ≥1.0) or low (<1.0) symptom level, and the CAT score was dichotomized as high (CAT ≥10) or low (CAT <10) symptom level [1]. All patients in the 2014 cohort were classified as GOLD 2017 groups A, B, C, or D based on frequent exacerbations and the dichotomized CAT score. Pharmacological treatment was presented as: no maintenance treatment, maintenance treatment with SAMA and/or LAMA; only LABA; LABA + LAMA/SAMA; only ICS; LABA + ICS or triple inhaled therapy with LAMA, LABA, and ICS. The reason for including SAMA in the category of anticholinergics was that the use of LAMA was not fully implemented in Sweden at the time of the first cohort. Instead, in 2005, many patients in Sweden were still using ipratropiumbromid three or four times daily as maintenance treatment. However, SAMA used only as rescue medication, or short-acting beta-2-agonists (SABA), were not included as maintenance treatment in our study. In the logistic regression analyses, three major recommended treatment alternatives were investigated: any bronchodilator therapy without ICS; LABA + ICS without LAMA; and triple inhaled therapy with LAMA, LABA, and ICS. The per-oral PDE-4-inhibitor roflumilast was not available at the time of the first survey in 2005 and was prescribed to only a few patients in the 2014 cohort, and subsequently was left out from the comparison.

### Statistics

Analyses were performed using SPSS version 22.0 (SPSS, Chicago). Cross-tabulation investigated patient characteristics by cohort year, and cross-tabulation and the chi-square test were used to investigate pharmacological maintenance treatment by cohort year stratified for sex and level of care, respectively. In the 2014 cohort, the three major recommended treatments bronchodilator therapy, LABA + ICS, and triple inhaled therapy were cross-tabulated according to GOLD 2017 groups A.B, C, and D, with repeated analyses stratified for sex and level of care. Multinomial regression was performed, both as a basic model with data available in both cohorts (basic 2005 and 2014 models) and as an extended model with data available in 2014 (extended 2014 model). The dependent variable included the three major treatment categories and no/other maintenance treatment [including only ICS and rescue medication with SABA or SAMA]. Independent variables in the basic model for comparison of the cohorts included sex, age (as a continuous variable), current daily smoking, level of education, underweight, overweight, obesity, frequent exacerbations or not, CCQ score (continuous variable), and being aware of a specific doctor responsible for the COPD treatment. The extended model of the 2014 cohort included the same independent variables with additional adjustment of heart disease, diabetes, and depression/anxiety, and with CAT score (continuous variable) replacing CCQ score. Independent variables with statistically significant associations in unadjusted multinominal analysis were included in the multivariate models. In the basic model for comparison, the analyses were performed stratified by cohort year and with interaction analysis using interaction terms for cohort year with each relevant variable with adjustment for the main effects and the potential confounding factors. A *p*-value of <0.05 was considered statistically significant. Due to the large number of groups, the Bonferroni equation of *α* / *n* = 0.05 was used to calculate the *p*-value for the stratified analyses of pharmacological treatments. As the number of treatment alternatives was seven, a *p*-value of 0.007 was considered statistically significant in these analyses.

### Ethics

The study was approved by the Regional Ethical Review Board in Uppsala (Dnr 2004: M-445 and Dnr 2010/090). Written informed consent was given by all the patients and health care professionals.

## Results

The patient characteristics of the two cohorts used in this study are shown in Table 1. The patients in the 2014 cohort were slightly older, less educated, and of a higher BMI, and less often had a specific doctor responsible for the treatment, but otherwise the cohorts were similar. Between 2005 and 2014, there was a statistically significant decrease in the proportions of patients treated with only ICS (from 5% to 2%), with only LABA + ICS (from 16% to 12%) and with no maintenance treatment (from 36% to 31%), and a statistically significant increase in the proportion of patients using triple inhaled therapy (from 29% to 40%) (Figure 1). The decrease in no maintenance treatment and ICS was only shown in primary care, while the decrease in LABA/ICS and the increase in triple therapy were statistically significant in both primary and secondary care (Table 2). Stratified by sex, the decrease in having no maintenance treatment, ICS, and ICS+LABA, and the increase in triple inhaled therapy were statistically significant only in women (Table 2). Within the group of patients treated with only muscarinic antagonists, the proportion of maintenance treatment with SAMA statistically significantly decreased from 45% to 3%. The change was the same regardless of level of care or sex (data not shown).

**Table 1.** Patient characteristics.

	2005 *N* (%)	2014 *N* (%)
**Sex**		
Male	451 (41%)	584 (44%)
Female	638 (59%)	745 (56%)
**Age**		
<60	242 (22%)	167 (13%)
60–69	5427 (48%)	553 (42%)
≥70	320 (30%)	609 (46%)
**Current daily smoking**		
No	779 (72%)	961 (74%)
Yes	308 (28%)	345 (26%)
**Education**		
Low	734 (69%)	1030 (79%)
High	333 (31%)	269 (21%)
**Body mass index**		
<20.0	117 (11%)	82 (6%)
20.0–24.9	368 (35%)	315 (24%)
25.0–29.9	362 (34%)	522 (41%)
≥30	206 (20%)	370 (29%)
**Symptom level**		
CCQ <1.0	748 (79%)	856 (77%)
CCQ ≥1.0	201 (21%)	249 (23%)
CAT <10	–	806 (66%)
CAT ≥10		423 (34%)
**Exacerbations in the last 6 months**		
0	673 (68%)	848 (66%)
≥1	368 (32%)	446 (34%)
Specific responsible doctor	524 (48%)	534 (42%)

Patient characteristics in respective cohort 2005 and 2014, reported as numbers and column percentages. High education denotes at least two years beyond the compulsory nine years of school in Sweden. CCQ = Clinical COPD Questionnaire; CAT = COPD Assessment Test.

**Table 2.** Treatment alternatives by level of care and sex.

	Primary care			Secondary care		
	2005	2014	*p*	2005	2014	*p*
No MT	310 (40%)	311 (35%)	0.029	79 (25%)	95 (22%)	0.281
LAMA/SAMA	76 (10%)	108 (12%)	0.137	32 (10%)	46 (11%)	0.874
LABA	7 (1%)	13 (2%)	0.301	4 (1%)	8 (2%)	0.546
LAMA/SAMA + LABA	20 (3%)	19 (2%)	0.541	11 (4%)	12 (3%)	0.556
ICS	45 (6%)	21 (2%)	<0.0001	14 (4%)	10 (2%)	0.097
LABA + ICS	130 (17%)	113 (13%)	0.017	49 (16%)	47 (11%)	0.051
LAMA + LABA + ICS	187 (24%)	308 (35%)	<0.0001	125 (40%)	218 (50%)	0.006
	Male			Female		
	2005	2014	*p*	2005	2014	*p*
No MT	165 (37%)	216 (37%)	0.894	224 (35%)	190 (26%)	<0.0001
LAMA/SAMA	50 (11%)	68 (12%)	0.780	58 (9%)	86 (12%)	0.
LABA	4 (1%)	9 (2%)	0.349	7 (1%)	12 (2%)	0.413
LAMA/SAMA + LABA	8 (2%)	11 (2%)	0.896	23 (4%)	20 (3%)	0.326
ICS	23 (5%)	17 (3%)	0.070	36 (6%)	14 (2%)	<0.0001
LABA + ICS	71 (16%)	72 (12%)	0.115	108 (17%)	88 (12%)	0.007
LAMA + LABA + ICS	130 (29%)	191 (33%)	0.181	182 (29%)	335 (45%)	<0.0001

Treatment with different medicines or combinations, in 2005 compared with 2014, distributed over sex and level of care. MT = maintenance treatment; LAMA = long-acting muscarinic antagonists; SAMA = short-acting muscarinic antagonists; LABA = long-acting beta-2-agonists; ICS = inhaled corticosteroids.

The number of patients with complete data on CAT score and exacerbations in 2014 was 1229. The distribution of these patients over GOLD groups was 21% in group A, 45% in group B, 3% in group C, and 31% in group D. Within group A, most patients had no maintenance treatment (52%), and within groups B, C, and D, triple inhaled therapy was the most common alternative (38%, 42%, and 59%, respectively) (Figure 2). This pattern remained, regardless of the level of care, with the exception that having no treatment and triple inhaled therapy were equally common (35%) in primary care patients within group B (Table 3). Among females, the pattern also remained, but in males, having no treatment was most common also in group B (39%).

**Table 3.** Major treatment alternatives in 2014 according to GOLD 2017 groups by level of care.

	A		B		C		D	
	PC	SC	PC	SC	PC	SC	PC	SC
No/other MT	54%	46%	35%	25%	27%	9%	19%	22%
Bronchodilator therapy	16%	16%	19%	17%	15%	13%	13%	15%
LABA + ICS	12%	12%	11%	13%	15%	8%	17%	11%
Triple inhaled therapy	18%	26%	35%	45%	43%	70%	51%	52%

Distribution of the three major recommended treatments over level of care. No/other includes patients with only rescue medication and with only ICS. Bronchodilator therapy includes maintenance treatment with long- or short-acting muscarinic antagonists and/or long-acting beta-2-agonists. GOLD = Global initiative for chronic Obstructive Lung Disease; PC = primary care; SC = secondary care; LABA = long-acting beta-2-agonists; ICS = inhaled corticosteroids; OR = odds ratio; CI = confidence interval; Ref = reference category

The results from the extended 2014 multinomial logistic regression model of the three major treatment alternatives with comparison of cohorts are shown in Table 4. In summary, bronchodilator therapy, LABA + ICS, and triple inhaled therapy were associated with a higher CAT score, no current smoking, and having a specific doctor responsible for COPD treatment. In addition, female sex was associated with bronchodilator and triple inhaled therapy, frequent exacerbations with LABA + ICS, and triple inhaled therapy, and an inverse association was found for overweight and obesity with bronchodilator therapy and for overweight with triple inhaled therapy. No statistically significant associations with comorbid conditions were found.

**Table 4.** Factors associated with recommended treatments in 2014.

	No/other	Bronchodilator therapy OR (95% CI)	*p*	LABA/ICS only OR (95% CI)	*p*	Triple inhaled therapy OR (95% CI)	*p*
Female sex	Ref	1.62	0.012	1.37	0.133	2.13	<0.0001
		(1.11 to 2.35)		(0.91 to 2.06)		(1.56 to 2.91)	
Age	Ref	1.03	0.100	1.00	0.803	1.02	0.180
		(1.00 to 1.06)		(0.97 to 1.03)		(0.99 to 1.04)	
High education	Ref	0.80	0.313	1.03	0.909	0.70	0.063
		(0.51 to 1.24)		(0.64 to 1.64)		(0.48 to 1.02)	
Underweight	Ref	0.45	0.109	1.58	0.297	1.02	0.966
		(0.17 to 1.20)		(0.67 to 3.73)		(0.50 to 2.09)	
Overweight	Ref	0.44	0.001	0.66	0.058	0.62	0.019
		(0.28 to 0.70)		(0.39 to 1.10)		(0.41 to 0.93)	
Obesity	Ref	0.56	0.025	0.79	0.405	0.72	0.140
		(0.34 to 0.93)		(0.45 to 1.39)		(0.47 to 1.11)	
Frequent exacerbations	Ref	1.31	0.254	2.02	0.004	2.34	<0.0001
		(0.83 to 2.06)		(1.26 to 3.25)		(1.62 to 3.36)	
Higher CAT score	Ref	1.03	0.040	1.04	0.002	1.07	<0.0001
		(1.00 to 1.05)		(1.02 to 1.07)		(1.05 to 1.09)	
Current daily smoking	Ref	0.64	0.039	0.37	<0.0001	0.40	<0.0001
		(0.42 to 0.98)		(0.23 to 0.62)		(0.28 to 0.57))	
Specific responsible doctor	Ref	2.77	<0.0001	1.70	0.014	1.95	<0.0001
		(1.89 to 4.05)		(1.12 to 2.60)		(1.41 to 2.69)	

Multinomial logistic regression of the three major recommended treatments. No/other includes patients with only rescue medication and with only ICS. Bronchodilator therapy includes maintenance treatment with long- or short-acting muscarinic antagonists and/or long-acting beta-2-agonists. LABA = long-acting beta-2-agonists; ICS = inhaled corticosteroids; CAT = COPD Assessment Test; OR = odds ratio; CI = confidence interval; Ref = reference category.

The basic multinomial regression 2014 model for comparison showed a similar pattern of associations as in the extended regression model for the 2014 cohort (data not shown). The stratification and interaction analyses showed that female sex was associated with triple inhaled therapy in 2014 but not in 2005 (*p* for interaction: 0.047) and that frequent exacerbations and CCQ score were associated with LABA + ICS in 2014 but not in 2005 (*p* for interaction: 0.046 and 0.033 respectively). Underweight was inversely associated with triple inhaled therapy in 2005 but not in 2014 (*p* for interaction: 0.048) and although having a specific doctor was statistically significantly associated with triple inhaled therapy in both 2005 and 2014, the association was weaker in 2014 (*p* for interaction: 0.031).

## Discussion

The first major finding of this multicenter observational study with both Swedish primary and secondary patients with a doctor’s diagnosis of COPD is that the pattern of maintenance treatment has changed during the last decade, with increased portions of triple inhaled therapy and decreased proportions of ICS, LABA + ICS, and no maintenance treatment. The second major finding is that, in 2014, frequent exacerbations, higher symptom level, female sex, and having a specific doctor responsible for treatment were the most important factors associated with the major maintenance treatment alternatives bronchodilator therapy, LABA + ICS, and triple inhaled therapy. Current daily smoking and higher BMI were associated with not receiving these treatments.

### Treatment patterns in 2005 and 2014

Between 2005 and 2014, the proportions of patients with no maintenance treatment and with only ICS have decreased in primary care. In both primary and secondary care, the proportion of treatment with LABA + ICS has decreased, and the proportion of triple inhaled therapy has increased. A possible explanation is increased indications for triple therapy, as the previous Swedish guidelines in 2005 were mainly based on spirometry rather than symptom staging. The changes could also be due to better implementations of COPD guidelines and better discrimination of the separate disease entities asthma and COPD, resulting in decreased ICS or LABA + ICS treatment without concomitant LAMA. The rationale for not treating COPD with ICS without concomitant LABA is well documented [7], and LAMA is the first choice of maintenance treatment with effects on both exacerbation risk and symptoms [1,22]. During the most recent years, the benefits of combining LAMA and LABA have been emphasized [23]. The low numbers of patients with double bronchodilation in our study could be due to the fact that this new treatment strategy had not yet been implemented by the time of our study. The Swedish national guidelines did not include the new GOLD assessment tool from 2011 until the most recent update in 2015 [24]. Interestingly, triple therapy was most common in all GOLD groups apart from group A in 2014 and was also the second most common alternative in group A. We speculate that the widespread use of triple inhaled therapy is due to the chronic characteristic of the disease, with a documented high proportion of persistent breathlessness in spite of maximum optimized treatment [25]. Other potential explanations may be increased availability of inhaled therapy in different devices and combinations, overtreatment due to heavy marketing from drug companies, and increased awareness and implementation of GOLD ABCD recommendations.

The pattern of COPD treatment has been investigated in two large US studies, where the majority of patients with severe lung-function impairment or a history of exacerbations still received no maintenance treatment [10,11]. In a UK primary care setting, COPD was also not treated according to GOLD and National Institute for Health and Care Excellence recommendations [15]. Many patients received no treatment despite experiencing symptoms, and among those on treatment most received ICS irrespective of the severity of airflow limitation, asthma diagnosis, and exacerbation history. Several other studies have reported undertreatment with bronchodilators in comparison with ICS [9,13], or a majority of patients receiving LABA/ICS without LAMA [16]. Rather few studies have investigated temporal change in treatment patterns, but our results of decreased proportions of patients with sole ICS treatment or no maintenance treatment at all over time are consistent with a study of COPD patients in UK primary care [12].

### Factors associated with recommended treatments

Our results that frequent exacerbations and higher symptom level are both associated with receiving bronchodilator therapy, LABA/ICS or triple inhaled therapy in 2014, are consistent with a previous study where these factors were major reasons for stepping up treatment [14]. However we found no previous study reporting our findings that smoking, BMI, a specific responsible doctor, and sex are associated with these treatment alternatives. We speculate that there is a ‘healthy smoker effect’ where current smoking is a marker for mild disease, as COPD patients with no symptoms and exacerbations do not need medication and are not motivated to quit smoking. Analogously, low BMI is a negative predictor in COPD [26], while overweight could be a marker for more stable disease. The fact that underweight was associated with triple therapy in 2005 and not in 2014 may be due to its covariance with more severe lung function, which was relatively more important for prescribing triple therapy in 2005. Having a specific doctor responsible for the COPD treatment should create better opportunities for prescribing medication. However, according to the stratification and interaction analyses of cohort year, access to a specific responsible doctor seems to be of somewhat less importance than in 2005, possibly due to the better implications of guidelines where all physicians are more aware of the present recommendations. The stronger associations of previous frequent exacerbations and higher symptom levels with LABA + ICS in 2014 than in 2005 may also be a consequence of changed and implemented guidelines.

The finding that female sex was associated with a higher likelihood of treatment with bronchodilators and triple inhaled therapy is most interesting. The prevalence of prescribed therapies in relation to sex has varied in previous studies. The PLATINO study showed higher proportions of maintenance treatment with inhaled bronchodilators and corticosteroids in women in a population-based study [27], but other studies have reported that COPD treatment is more common in men [16,28].

Women are known to be more susceptible to developing COPD [29] and have less declining COPD-related mortality rates than men [30], but factors influencing sex differences in treatment are still an understudied area. Women have been reported to have more symptoms and more exacerbations in COPD [31], but as our analyses adjusted for both CAT and CCQ scores and for previous frequent exacerbations, difference in these factors should not explain the increased medication in women in 2014. Finally, as our questionnaire assessed actual treatment and not just prescription, adherence to treatment could influence the proportion of patients with maintenance treatment. Yet, adherence and correct inhaler technique have been reported to be equal between sexes [32] or even better in men [33,34] and subsequently should not explain our results. In summary, more research in sex differences and its implications for therapy is obviously needed [35,36]. However, we speculate that the association of female sex with several treatments in 2014 and not in 2005 could be explained by a higher awareness among physicians of the increased COPD prevalence, mortality and symptom level in women [37].

### Strengths and limitations

The major strengths of our study are that it is a multicenter real-world study with patients from both primary and secondary care, which should ensure a high level of external validity and generalizability, and that the analyses include two cohorts with 10 years’ difference in time.

One important potential limitation is that the inclusion criterion of the patient population is a doctor’s diagnosis of COPD. In the present study, only patient questionnaires were used, and the COPD diagnoses were not confirmed by record review of spirometry, which means that our patient population could potentially include patients with an incorrect COPD diagnosis [21]. However, our purpose was to perform a real-life study of patients with a doctor’s diagnosis of COPD, and spirometry is unfortunately not always performed in clinical praxis. Using questionnaire data without spirometry-confirmed diagnoses reflects clinical reality. Another limitation with the lack of lung-function data is that the spirometric staging could also be an important factor influencing the choice of pharmacological treatment. However, our main purpose was to investigate the influence of the GOLD 2017 ABCD groups based on exacerbation frequency and level of symptoms. The proper GOLD definition of frequent exacerbations includes at least two exacerbations recent 12 months [1], but since our data were based on the exacerbation frequency in the previous 6 months we chose to define frequent exacerbations as at least one in the previous 6 months. The characterization and the basic multinomial logistic regression models for comparison of the patient cohorts included symptom level assessed by CCQ, but the GOLD ABCD staging and the extended multinomial logistic regression of the 2014 cohort included the health status measurement CAT. The reason for this procedure was that CAT now is regarded as the first choice for symptom evaluation in the group ABCD assessment, but was not available in 2005 and subsequently could not be used for comparison of the cohorts. However, CAT and CCQ correlate very well and can be used as substitutes for each other [38].

## Conclusion

During the last decade, the proportion of COPD patients with triple inhaled therapy has increased, and having no maintenance treatment, only ICS or only LABA/ICS have decreased. Triple inhaled therapy is the most common treatment combination in GOLD groups B, C, and D. The most important factors associated with having triple inhaled therapy in COPD are previous exacerbations, lower health status, female sex, and having a specific doctor responsible for the COPD treatment.

## Geolocation information

The study was performed in central Sweden.
